# Depression, anxiety in adolescents with exogenous obesity and depression, anxiety, caregiver burden, and burnout in their mothers

**DOI:** 10.3389/fped.2025.1632927

**Published:** 2025-07-31

**Authors:** Can Celiloğlu, İhsan Turan, Perihan Çam Ray, Semine Özdemir Dilek, Mevra Çay, Şükriye Tuğçe Çelebi, Ayşe Merve Çimen, Zeynep Namlı, Bilgin Yüksel

**Affiliations:** ^1^ Department of Pediatric Endocrinology, Çukurova University Faculty of Medicine, Adana, Türkiye; ^2^ Department of Child and Adolescent Psychiatry, Çukurova University Faculty of Medicine, Adana, Türkiye; ^3^ Department of Psychiatry, Çukurova University Faculty of Medicine, Adana, Türkiye

**Keywords:** obesity, maternal burden, depression, anxiety, burnout

## Abstract

**Introduction:**

The development of obesity is associated with various biological, environmental, and psychological factors, and there is a bidirectional interaction between obesity and psychological health. Several reports have highlighted the intrafamilial transmission of psychopathologies among individuals with obesity. This study aimed to assess symptoms of depression and anxiety in adolescents with exogenous obesity and their mothers, along with maternal caregiver burden and burnout levels.

**Methods:**

Adolescents diagnosed with exogenous obesity and their mothers were prospectively enrolled in the study. Clinical data were recorded, and participants completed standardized assessment tools: children filled out the Screen for Child Anxiety Related Emotional Disorders (SCARED) and the Kovacs Children's Depression Inventory (CDI), while mothers completed the Beck Depression Inventory (BDI), Beck Anxiety Inventory (BAI), Maslach Burnout Inventory (MBI), and Zarit Caregiver Burden Scale. Results were compared with those of a control group.

**Results:**

A total of 49 obese adolescents (aged 12–18) and their mothers, and 43 age- and sex-matched controls with their mothers, were evaluated. The mean BMI of the obesity group was 35.29 ± 4.77 kg/m^2^ (BMI-SDS: 2.44 ± 0.40). No significant differences were observed between adolescents with obesity and control subjects regarding depression and anxiety scores. Similarly, mothers’ depression, anxiety, and caregiver burden scores did not significantly differ. However, emotional exhaustion and depersonalization subscale scores on the MBI were significantly higher among mothers of adolescents with obesity.

**Discussion:**

All adolescents showed elevated depression and anxiety scores compared to national averages, regardless of obesity status. Levels of emotional exhaustion and depersonalization were significantly elevated in mothers of adolescents with obesity. Assessing maternal burnout may contribute to more effective management of childhood obesity.

## 1 Introduction

Childhood obesity is generally caused by decreased physical activity and/or improper nutrition leading to excessive energy intake (1). Pharmacological interventions play a limited role in obesity treatment; the primary components of therapy include dietary modifications, exercise, and behavioral changes (1). Adolescence is a developmental stage marked by heightened stress reactivity, emotional sensitivity, and vulnerability (2). Obesity-related psychosocial stress may further intensify stress during adolescence (3, 4). Cumulative stress can negatively impact mental health (5). Many obese children experience lower academic performance, reduced quality of life, diminished self-esteem, and negative body image (6). Symptoms commonly observed in depressive disorders, such as impaired concentration and low energy, may interfere with participation in physical activity, potentially hindering obesity treatment and contributing to worsening severity (7). The relationship between depression and obesity is bidirectional, with each condition increasing the risk of the other (8).

Although it is generally accepted that childhood obesity has adverse effects on mental health, the literature presents conflicting results regarding the association. A study conducted in Iran reported no significant difference in the prevalence of depression among obese adolescents. It was suggested that in Iranian culture, being overweight is associated with health and happiness, possibly explaining the lack of increased depression (9). Zakeri et al. also found that obesity did not increase the incidence of emotional problems, highlighting the role of cultural factors (10).

Previous studies have explored the possibility that psychopathologies observed in obese individuals may be transmitted within families. One study reported a significant association between psychological problems in obese children and those in their mothers (6). A review of nine studies from the United States and Europe found that maternal chronic depression may be a risk factor for childhood obesity (11). However, there are also reports suggesting that childhood obesity is not necessarily linked to maternal depression, negative life events, poor overall family functioning, or ineffective parenting (12). A study conducted in Turkey found significantly increased anxiety but not depression in mothers of obese children, suggesting that impaired family functioning may negatively impact the treatment process (13). Emotional and physical support from parents plays a crucial role in adolescents' weight-loss efforts (14).

Burnout is an emotional state resulting from cumulative exposure to chronic occupational and life stress, characterized by emotional exhaustion, physical fatigue, and cognitive weariness. The Maslach Burnout Inventory (MBI) is the most widely used tool to assess burnout (15). To date, there is no study in the literature investigating burnout in families of obese children. Maternal depression may lead to emotional detachment from the child's weight problems, delays in seeking medical help, and poor adherence to treatment. We hypothesized that maternal burnout may be more prevalent among mothers of obese adolescents. Since obesity is a chronic condition, we also evaluated the “burden” of adolescent obesity on their caregivers.

## 2 Materials and methods

This study was designed as a cross-sectional, relational screening model using convenience sampling. Between June 2021 and June 2022, adolescents aged 12–18 years who presented to the Pediatric Endocrinology outpatient clinic at Çukurova University Faculty of Medicine for obesity were randomly selected and evaluated through physical examination and structured interview forms. The control group consisted of adolescents of the same age range and their mothers who visited the general pediatrics outpatient clinic and had no obesity or chronic illnesses. All interviews were conducted face-to-face by a pediatrician. The diagnosis of obesity was based on a body mass index (BMI) greater than +2 standard deviations (SDS) for age (16). The study received ethical approval from the Non-Interventional Clinical Research Ethics Committee of Çukurova University (Approval Date: 11/06/2021; Approval No: 112). The study was conducted in accordance with the principles of the Declaration of Helsinki. Written informed consent for participation in the study and for the publication of the results was obtained from all legal guardians.

### 2.1 Inclusion criteria

- Adolescents aged 12–18 years with a BMI greater than +2 SDS for age.
- Complete responses to all administered scales.

### 2.2 Exclusion criteria

- Presence of intellectual disability, psychotic disorder, bipolar disorder, or autism in the adolescent or mother.
- Syndromic or endogenous obesity (e.g., Cushing syndrome, Alström syndrome).
- Musculoskeletal disorders limiting movement.
- Presence of systemic illnesses other than metabolic syndrome.

### 2.3 Psychometric assessments

#### 2.3.1 Psychometric assessments administered to mothers

Maternal depressive symptoms were assessed using the Beck Depression Inventory (BDI), a self-report scale where higher scores indicate greater severity of depression (17, 18). Maternal anxiety symptoms were assessed with the Beck Anxiety Inventory (BAI), also a self-report scale, where higher scores reflect higher anxiety levels (19, 20). Burnout was evaluated using the Maslach Burnout Inventory (MBI), which includes subscales for emotional exhaustion, depersonalization, and reduced personal accomplishment (21, 22). Higher scores on emotional exhaustion and depersonalization, and lower scores on personal accomplishment, indicate higher burnout levels (23). Caregiver burden was measured using the Zarit Caregiver Burden Scale, where higher scores reflect greater burden (24, 25).

#### 2.3.2 Psychometric assessments administered to adolescents

Adolescents' anxiety was evaluated using the Screen for Child Anxiety Related Emotional Disorders (SCARED), with higher scores indicating more severe anxiety (26, 27). The scale developed by Kovacs (CDI) was used to score depressive symptoms in adolescents where a high total score indicates high level of depression (28, 29).

### 2.4 Statistical analysis

Data were analyzed using the Statistical Package for the Social Sciences (SPSS) version 16. Descriptive statistics were presented as percentages (%) for categorical variables, means ± standard deviation for normally distributed data, and medians for non-normally distributed data. Group comparisons were performed using the Chi-square test for non-parametric variables and the independent samples t-test for parametric variables. A *p*-value <0.05 was considered statistically significant.

## 3 Results

A total of 49 obese adolescents and 43 controls aged between 12 and 18 years were evaluated. The mean age of the obese group was 14.34 ± 1.66 years (min: 12, max: 18), while that of the control group was 15.00 ± 1.58 years (min: 12, max: 18) (*p* = 0.06). Among the obese participants, 33 were female and 16 were male; in the control group, 28 were female and 15 were male. There was no significant difference in sex distribution between the groups (*p* = 0.82) (Table 1).

**Table 1:** Demographic and psychometric measures of obese and control groups.

Variables	Obese group (*n* = 49)	Control group (*n* = 43)	*p*-value
Age (years)	14,34 ± 1,66	15,00 ± 1,58	0,06
Sex			
Female	*n* = 33 (%….)	*n* = 28 (%….)	0,82
Male	*n* = 16 (%….)	*n* = 15 (%….)	
Body mass index (BMI) (kg/m^2^)	35,29 ± 4,77	22,45 ± 3,21	<0,05
BMI-SDS	2,44 ± 0,40	0,62 ± 0,67	<0,05
Family history of obesity	*n* = 35 (%79,5)	*n* = 9 (% 20,5)	<0,01
Maternal education (years)	8,04 ± 3,69	7,90 ± 4,36	0,87
Maternal BMI	31,07 ± 0,4	26,6 ± 4,3	<0,05
Adolescent depression score	16,36 ± 6,96	15,39 ± 9,69	0,57
Adolescent anxiety score	29,95 ± 14,09	27,04 ± 14,35	0,32
Maternal depression score	15,08 ± 10,5	15,53 ± 11,38	0,84
Maternal anxiety score	17,24 ± 13,13	15,14 ± 11,48	0,41
Caregiver burden score	24,81 ± 15,1	20,62 ± 10,82	0,13
Emotional exhaustion (Maslach)	9,14 ± 5,397	6,23 ± 4,659	<0,05
Depersonalization (Maslach)	3,14 ± 2,94	1,74 ± 1,63	<0,05

The mean body mass index (BMI) of the obese group was 35.29 ± 4.77 kg/m^2^ and the BMI-SDS was 2.44 ± 0.40, whereas the control group had a mean BMI of 22.45 ± 3.21 kg/m^2^ and a BMI-SDS of 0.62 ± 0.67 (*p* < 0.01). The mean total depression score among obese adolescents was 16.36 ± 6.96 and the total anxiety score was 29.95 ± 14.09, while these values were 15.39 ± 9.69 and 27.04 ± 14.35, respectively, in the control group. No statistically significant difference was found between the groups (*p* = 0.57 and *p* = 0.32, respectively) (Table 1).

The mean maternal BMI in the study group was 31.07 ± 0.4, compared to 26.6 ± 4.3 in the control group (*p* = 0.01). The mean years of maternal education were 8.04 ± 3.69 years in the obese group and 7.9 ± 4.3 years in the control group, with no significant difference (*p* = 0.87). Among the mothers of the obese group, the depression score was 15.08 ± 10.5 (median: 14), anxiety score was 17.24 ± 13.13 (median: 16), and caregiver burden score was 24.81 ± 15.15. In the control group, these scores were 15.53 ± 11.38, 15.14 ± 11.48, and 20.62 ± 10.82, respectively. None of the differences were statistically significant (*p* = 0.84, *p* = 0.41, *p* = 0.13, respectively) (Table 1).

Among mothers of obese adolescents, the mean scores on the Maslach Burnout Inventory were as follows: emotional exhaustion: 9.14 ± 5.39, depersonalization: 3.14 ± 2.94. In the control group, these scores were 6.23 ± 4.65 and 1.74 ± 1.63, respectively (*p* = 0.007 for both). Emotional exhaustion and depersonalization scores were significantly higher among mothers in the obese group. The personal accomplishment subscale score was 7.87 ± 5.68 in the study group and 6.81 ± 5.75 in the control group, with no significant difference (*p* = 0.376) (Table 1).

Due to exclusion criteria, individuals or guardians with a diagnosed psychiatric disorder were not included. At the time of enrollment, none of the adolescents in the study group were receiving psychiatric or other medical treatment. All participants were within the age range of compulsory education, and none had dropped out of school.

## 4 Discussion

Previous studies from Turkey have reported elevated depression and anxiety scores in obese adolescents (30, 31). However, our study did not identify significant differences in depression or anxiety scores between obese and non-obese adolescents. A report from Iran, a country geographically and culturally similar to Turkey, suggested that obesity may serve as a protective factor against psychosocial disorders (10). This has been attributed to cultural perceptions of overweight individuals as being healthier.

Studies conducted in Turkey have reported average childhood depression inventory (CDI) scores of 11.3 ± 6.02 and 13.3 ± 7.3 (32, 33). In our study, CDI scores were elevated in both obese and non-obese adolescents compared to those reported in the literature. These elevated depression scores may be related to the COVID-19 pandemic period during which the study was conducted. Supporting evidence from Turkey indicates that mental health outcomes worsened during this time (34). Similarly, previous studies using the Screen for Child Anxiety Related Emotional Disorders (SCARED) in Turkey reported a mean control group anxiety score of 26.7 ± 10.6, which is lower than our findings (35), suggesting a general elevation in anxiety possibly due to the pandemic.

We found no significant differences in maternal depression and anxiety scores between the groups. Some studies have reported higher maternal depression in obese groups, while others have found no difference (11, 12). Our findings align with previous research from Turkey indicating no significant difference in maternal depression scores (13). The significantly higher maternal BMI in the obese group is consistent with earlier literature (36). It is well established that both genetic and environmental components contribute to familial predisposition to obesity. Although our study did not focus specifically on “obesogenic” behavioral patterns within families—such as the nutritional content of snacks, portion sizes, chewing duration, or exposure to digital screens during meals—these factors may represent important areas for future research.

The mean caregiver burden score among mothers of obese adolescents was 24.81 ± 15.1, a value classified as “mild burden” (range: 22–46) according to the literature (37). While caregiver burden was elevated in mothers of obese adolescents, the difference was not statistically significant compared to controls. This scale has not previously been applied to non-syndromic obesity. Given the chronic nature of obesity and its treatment, emotional exhaustion in mothers of obese adolescents may mask symptoms of depression and anxiety. Furthermore, maternal depersonalization may result in underreporting or under-perception of psychological distress and caregiver burden.

One of the strengths of this study is the face-to-face, expert-conducted evaluations of both adolescents and their mothers. To our knowledge, the use of the Maslach Burnout Inventory among mothers of obese adolescents has not been previously reported. Emotional exhaustion and depersonalization scores were significantly higher in the mothers of obese adolescents. This burnout may be a cause or a consequence of adolescent obesity. Mothers who are also obese may feel powerless to change the situation, and a perceived lack of benefit from medical interventions may contribute to emotional exhaustion. Addressing maternal hopelessness may yield positive outcomes in adolescent obesity management.

### 4.1 Limitations and future research

This study has several limitations, including a relatively small sample size, cross-sectional design, reliance on self-report scales to assess psychological symptoms, and the potential impact of the COVID-19 pandemic on mental health. Given that mothers have traditionally played a significant role in shaping children's food preferences and energy intake through direct interaction and regulation of the feeding environment, they were included in our assessment. However, contemporary shifts in caregiving roles necessitate the evaluation of fathers as well, and psychometric studies focusing on paternal involvement may provide valuable and novel insights. The relationship between burnout and adherence to medical recommendations, as well as the emotional transmission of burnout from caregiver to child, warrants further investigation. Interventional studies evaluating the effects of cognitive-behavioral support for caregivers on treatment outcomes may also be beneficial. Longitudinal studies with larger sample sizes are recommended to further explore the potential bidirectional relationship between childhood obesity, maternal obesity, and caregiver burnout.

## 5 Conclusion

Although depression and anxiety scores were elevated in all adolescents compared to national norms, no significant association with obesity was identified. Emotional exhaustion and depersonalization were significantly higher among mothers of obese adolescents. Assessing and supporting maternal burnout may contribute positively to adolescent obesity management. Due to the cross-sectional design, no causal inferences can be drawn. Future longitudinal research is needed to clarify the potential bidirectional relationship between childhood obesity, maternal obesity, and caregiver burnout.

## Data Availability

The datasets presented in this study can be found in online repositories. The names of the repository/repositories and accession number(s) can be found below: https://docs.google.com/spreadsheets/d/17YL95YeJ1eb71C07Y1wpl3vvM7hadq9R/edit?gid=992405784#gid=992405784.

## References

[1] Koyuncuoğlu Güngör N. Overweight and obesity in children and adolescents. J Clin Res Pediatr Endocrinol. (2014) 6(3):129–43. 10.4274/jcrpe.1471 25241606 PMC4293641

[2] Guyer AE Silk JS Nelson EE. The neurobiology of the emotional adolescent: from the inside out. Neurosci Biobehav Rev. (2016) 70:74–85. 10.1016/j.neubiorev.2016.07.037 27506384 PMC5074886

[3] Puhl RM Peterson JL Luedicke J. Weight-based victimization: bullying experiences of weight loss treatment–seeking youth. Pediatrics. (2013) 131:e1–9. 10.1542/peds.2012-1106 23266918

[4] Kiess W. Obesity. In: Dattani MT Brook CGD, editors. Brook’s Clinical Pediatric Endocrinology. Oxford: John Wiley & Sons Ltd. (2020). p. 701–29.

[5] Slavich GM Toussaint L. Using the stress and adversity inventory as a teaching tool leads to significant learning gains in two courses on stress and health. Stress and Health. (2014) 30:343–52. 10.1002/smi.2523 23955924 PMC4361060

[6] Roth B Munsch S Meyer A Isler E Schneider S. The association between mothers’ psychopathology, childrens’ competences and psychological well-being in obese children. Eat Weight Disord. (2008) 13:129–36. 10.1007/BF03327613 19011370

[7] Dinas PC Koutedakis Y Flouris AD. Effects of exercise and physical activity on depression. Ir J Med Sci. (2011) 180:319–25. 10.1007/s11845-010-0633-9 21076975

[8] Milaneschi Y Simmons WK van Rossum EFC Penninx BW. Depression and obesity: evidence of shared biological mechanisms. Mol Psychiatry. (2019) 24:18–33. 10.1038/s41380-018-0017-5 29453413

[9] Roohafza H Kelishadi R Sadeghi M Hashemipour M Pourmoghaddas A Khani A. Are obese adolescents more depressed? J Educ Health Promot. (2014) 3:74. 10.4103/2277-9531.134908 25077167 PMC4113990

[10] Zakeri M Sedaghat M Motlagh ME Tayari Ashtiani R Ardalan G. BMI correlation with psychiatric problems among 10–18 years Iranian students. Acta Med Iran. (2012) 50:177–84. 22418986

[11] Lampard AM Franckle RL Davison KK. Maternal depression and childhood obesity: a systematic review. Prev Med (Baltim). (2014) 59:60–7. 10.1016/j.ypmed.2013.11.020 PMC4172574 24291685

[12] Gibson LY Byrne SM Davis EA Blair E Jacoby P Zubrick SR. The role of family and maternal factors in childhood obesity. Med J Aust. (2007) 186:591–5. 10.5694/j.1326-5377.2007.tb01061.x 17547550

[13] Akay AP Ozturk Y Avcil SN Kavurma C Tufan E. Relationships between pediatric obesity and maternal emotional states and attitudes. Int J Psychiatry Med. (2015) 50:178–90. 10.1177/0091217415605032 26359289

[14] Jensen CD Duraccio KM Hunsaker SL Rancourt D Kuhl ES Jelalian E A qualitative study of successful adolescent and young adult weight losers: implications for weight control intervention. Child Obes. (2014) 10:482–90. 10.1089/chi.2014.0062 25369460

[15] Frajerman A Morvan Y Krebs M-O Gorwood P Chaumette B. Burnout in medical students before residency: a systematic review and meta-analysis. Eur Psychiatry. (2019) 55:36–42. 10.1016/j.eurpsy.2018.08.006 30384110

[16] Neyzi O Günöz H Furman A Bundak R Gökçay G Darendeliler F Türk çocuklarında vücut ağırlığı, boy uzunluğu, baş çevresi ve vücut kitle indeksi referans değerleri. Çocuk Sağlığı ve Hastalıkları Dergisi. (2008) 51:1–14.

[17] Beck AT. An inventory for measuring depression. Arch Gen Psychiatry. (1961) 4:561. 10.1001/archpsyc.1961.01710120031004 13688369

[18] Hisli N. Beck depresyon envanterinin geçerliliği üzerine bir çalışma. Turk J Psychol. (1987) 6:118–22.

[19] Beck AT Epstein N Brown G Steer RA. An inventory for measuring clinical anxiety: psychometric properties. J Consult Clin Psychol. (1988) 56:893–7. 10.1037/0022-006X.56.6.893 3204199

[20] Ulusoy M Şahin NH Erkmen H. Turkish version of the Beck anxiety inventory: psychometric properties. J Cogn Psychother. (1998) 12:163–72.

[21] Maslach C Jackson SE. The measurement of experienced burnout. J Organ Behav. (1981) 2:99–113. 10.1002/job.4030020205

[22] Ergin C. Doktor ve Hemşirelerde Tükenmişlik ve Maslach Tükenmişlik Ölçeğinin Uyarlanması. In: Bayraktar R Dağ İ, editors. VII. Ulusal Psikoloji Kongresi Bilimsel Çalışmaları. Ankara: Türk Psikologlar Derneği (1992). p. 1–278.

[23] Maslach C Jackson SE. Maslach Burnout Inventory. 2nd ed. Palo Alto: Consulting Psychologists Press (1986). p. 1–34.

[24] Zarit SH Reever KE Bach-Peterson J. Relatives of the impaired elderly: correlates of feelings of burden. Gerontologist. (1980) 20:649–55. 10.1093/geront/20.6.649 7203086

[25] Özlü A Yıldız M Aker T. Zarit bakıcı yük ölçeğinin şizofreni hasta yakınlarında geçerlilik ve güvenilirlik çalışması. Nöropsikiyatri Arşivi. (2009) 46:38–42.

[26] Birmaher B Brent DA Chiappetta L Bridge J Monga S Baugher M. Psychometric properties of the screen for child anxiety related emotional disorders (SCARED): a replication study. J Am Acad Child Adolesc Psychiatry. (1999) 38:1230–6. 10.1097/00004583-199910000-00011 10517055

[27] Karaceylan Çakmakçı F. Çocuklarda Anksiyete Bozukluklarını Tarama Ölçeği Geçerlik ve Güvenirlik çalışması. Kocaeli: Kocaeli University (2004).

[28] Öy B. Çocuklar için depresyon ölçeği: geçerlilik ve güvenirlik çalışması. Turk Psikiyatri Dergisi. (1991) 2:132–5.

[29] Kovacs M. Rating scales to assess depression in school-aged children. Acta Paedopsychiatr. (1981) 46:305–15. 7025571

[30] Ardic A. Fazla kilolu ve obez adolesanların depresyon anksiyete ve stres düzeyleri. Turk J Fam Med Primary Care. (2020) 14:384–90. 10.21763/tjfmpc.665955

[31] Erermis S Cetin N Tamar M Bukusoglu N Akdeniz F Goksen D. Is obesity a risk factor for psychopathology among adolescents? Pediatr Int. (2004) 46:296–301. 10.1111/j.1442-200x.2004.01882.x 15151546

[32] Demir T Karacetin G Demir DE Uysal O. Epidemiology of depression in an urban population of Turkish children and adolescents. J Affect Disord. (2011) 134:168–76. 10.1016/j.jad.2011.05.041 21683451

[33] Yılmazel GGO. Self-esteem and depression levels among 12–17 years old students in Kargı–Çorum. J Health Sci. (2012) 21:20–9.

[34] Çelik E Gezmen Karadağ M Bayazit AD. COVİD-19 pandemi dönemi sürecinde adölesanlarin ekran kullanimi, depresyon durumlari ve antropometrik ölçümlerindeki değişimin değerlendirilmesi. Gazi Sağlık Bilimleri Dergisi. (2022) (Special issue):7–13. 10.52881/gsbdergi.1082594

[35] Karaca Ünlü A Ağbaş A Aksu B Elevli M. Primer enürezis nokturna tanili Çocuklar ve annelerinde anksiyete düzeyinin değerlendirilmesi. Istanbul Tip Fakültesi Dergisi. (2020) 83:100–4. 10.26650/IUITFD.2018.0031

[36] Zeller MH Hunsaker S Mikhail C Reiter-Purtill J McCullough MB Garland B Family factors that characterize adolescents with severe obesity and their role in weight loss surgery outcomes. Obesity. (2016) 24:2562–9. 10.1002/oby.21676 27753228 PMC5379472

[37] Atagün M Balaban O Atagun Z Elagoz M Ozpolat A. Caregiver burden in chronic diseases. Curr Approach Psychiatry. (2011) 3(3):513–52. 10.5455/cap.20110323

